# Supplementary material for: Influence of drought stress on afalfa yields and nutritional composition
Source: BMC Plant Biol. 2018 Jan 15;18:13. doi: 10.1186/s12870-017-1226-9 (PMC5769550; doi:10.1186/s12870-017-1226-9)
Supplement: Supplementary file 1 [file 12870_2017_1226_MOESM1_ESM.docx]

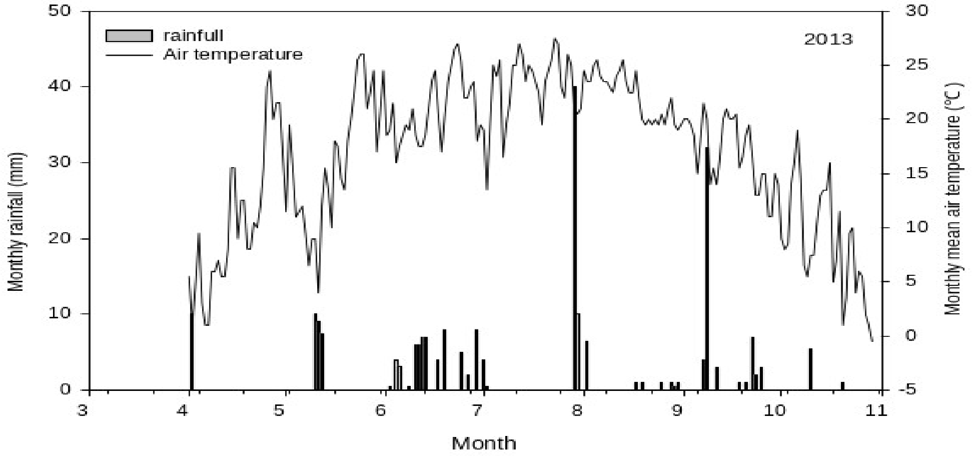

rainfull
Air temperature
2013
Monthly rainfall (mm)
Monthly mean air temperature (℃)
Month

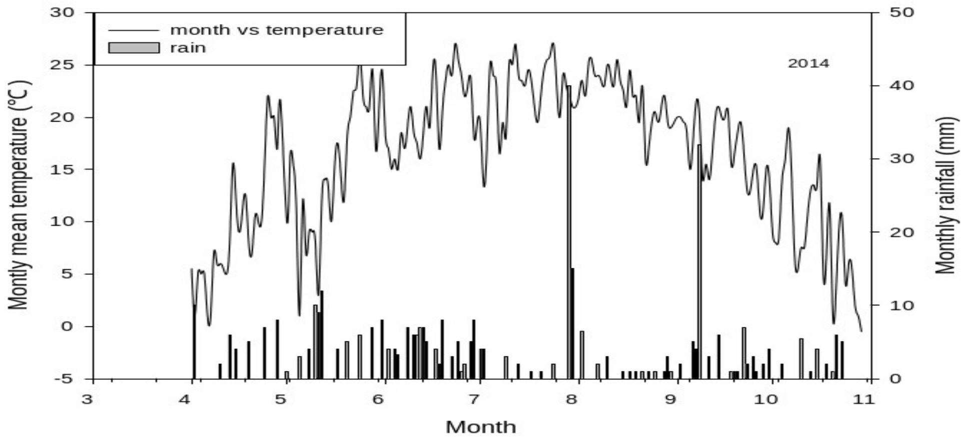

month vs temperature
rain
2014
Montly mean temperature (℃)
Monthly rainfall (mm)
Month
